# A successful pregnancy in a patient with secondary hypertension caused by adrenal adenoma: a case report

**DOI:** 10.1186/s12884-019-2262-2

**Published:** 2019-04-03

**Authors:** Xin Zhang, Hang Liao, Xiaojiang Zhu, Di Shi, Xiaoping Chen

**Affiliations:** 1 Department of cardiology, West China Hospital, Sichuan University, Chengdu, China; 2 Naidong People’s Hospital, Shannan, Tibet Autonomous Region China

**Keywords:** Hypertension, Cushing’s syndrome, Adrenal adenoma, Pregnancy

## Abstract

**Background:**

Secondary hypertension is a rare complication in pregnancy that causes poor outcomes, such as preeclampsia, premature delivery, intrauterine growth retardation, stillbirths, spontaneous abortion or intrauterine death. Cushing’s disease caused by an adrenal adenoma is rare during pregnancy and may be overlooked by obstetricians and physicians, but can lead to hypertension, diabetes mellitus and an increased risk of fetal and maternal morbidity. Approximately 200 cases have been reported in the literature. Here, we report the successful management of a pregnant patient with Cushing’s syndrome due to an adrenal adenoma.

**Case presentation:**

The 35-year-old Chinese female had no individual or family medical history of hypertension, and did not exhibit chronic kidney disease, diabetes mellitus, autoimmune and common endocrine diseases. Her blood pressure was elevated from the 16th week of gestation and was not controlled by 30 mg nifedipine twice a day. Examination in our department revealed her 24 h urinary free cortisol (24 h UFC) level was 1684.3 μg/24 h (normal range: 20.26–127.55 μg/24 h) and plasma adrenocorticotropic hormone was < 1.00 ng/L in three independent measurements (normal range: 5–78 ng/L). Ultrasonography demonstrated a mass (2.9 cm × 2.8 cm) in the right side of the adrenal gland. Magnetic resonance imaging without contrast showed a 3.2 cm diameter mass in the right-side of the adrenal gland. Other medical tests were normal. Laparoscopic adrenalectomy was performed at the 26th week of gestation by a urological surgeon in the West China Hospital. Histopathology revealed an adrenocortical adenoma. After surgery, the patient accepted glucocorticoid replacement therapy. The remaining trimester continued without complication and her blood pressure was normal at the 32nd week of gestation without antihypertensive therapy. The patient gave birth to a healthy boy at the 40th week of gestation.

**Conclusions:**

Cushing’s syndrome caused by adrenal adenoma is rare during pregnancy. This unique case suggested that analysis of the UFC level and circadian rhythm of plasma cortisol provides a suitable strategy to diagnose Cushing’s syndrome during pregnancy. Laparoscopic surgical resection in the second trimester provides a reasonable approach to treat pregnant patients exhibiting Cushing’s syndrome caused by an adrenal adenoma.

## Background

Chronic hypertension is a common medical complication in pregnancy that is defined as hypertension present before pregnancy or the 20th week of gestation [1]. Chronic hypertension during pregnancy can be classified as primary (90%) and secondary (10%) hypertension [2]. The causes of secondary hypertension in pregnancy include chronic kidney disease, renovascular hypertension, pheochromocytoma, primary aldosteronism and Cushing’s syndrome [3]. All of these diseases may lead to poor pregnancy outcomes, such as preeclampsia and premature delivery, intrauterine growth retardation, stillbirths, spontaneous abortion or intrauterine death [3].

Cushing’s syndrome during pregnancy is rare and may be overlooked by cardiovascular physicians. The first case-report of pregnancy with Cushing’s syndrome was by Hunt and McConahey in 1953 [4]. To date, nearly 200 cases of Cushing’s syndrome during pregnancy have been reported in the literature [5]. The current case is the first report of Cushing’s syndrome caused by adrenal adenoma during a pregnancy in Western China, which has a population of approximately 380 million. Cushing’s syndrome is a systemic disorder caused by exposure to excess glucocorticoids, which impair the endocrine system [6]. Cushing’s syndrome can lead to hypertension and diabetes mellitus and can increase the risk of maternal and fetal morbidity [7]. Here, we report the successful management of a pregnant patient with Cushing’s syndrome caused by an adrenal adenoma, which was diagnosed during pregnancy, and we review relevant literature.

## Case presentation

### General information

**Fig. 1:**
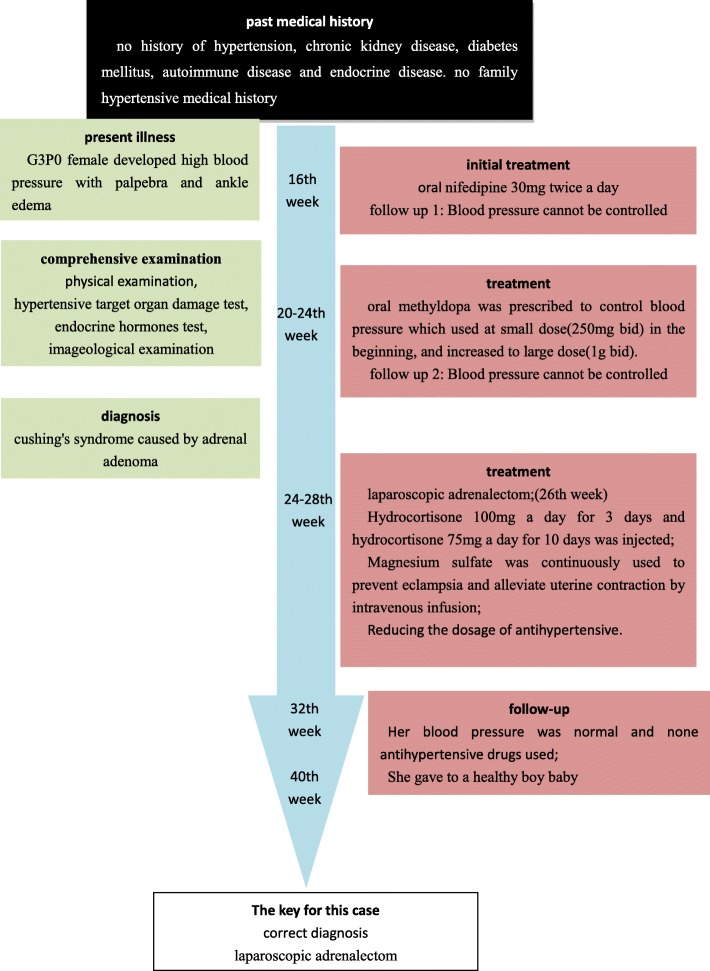
Timeline of the patient care

The patient was a 35-year-old married Chinese female (G3P0). The timeline of patient care is shown in Fig. 1.

### Presented illness

The patient developed high office blood pressure (approximately 159/96 mmHg) from the 16th week of gestation. Unfortunately, her blood pressure continued to rise (maximal at approximately 180/110 mmHg) as gestation progressed, combined with palpebra and ankle edema. The high blood pressure could not be controlled by 30 mg nifedipine twice a day. She was admitted to the Cardiovascular Department of West China Hospital, Sichuan University at the 20th week of gestation exhibiting hypertension.

### Past medical information

The patient had no individual or family medical history of hypertension. She did not exhibit chronic kidney disease, diabetes mellitus, or autoimmune disease. Medical information before gestation showed that the patient was healthy and without any obvious endocrine disease.

### Admission information

At the 20th week of gestation, the patient was admitted to the Cardiovascular Department, West China Hospital, Sichuan University. The patient underwent a comprehensive medical examination. Her office blood pressure was 168/100 mmHg, weight was 68 kg, height was 162 cm, and body mass index was 25.9 kg/m^2^. The blood pressure in her left and right upper extremities was 168/100 mmHg and 166/98 mmHg, respectively, and in her left and right lower extremities was 180/110 mmHg and 184 /112 mmHg, respectively. The ratio of blood pressure for the upper and lower extremities was normal. The patient had no pulmonary edema and no cerebral or visual symptoms. Her baseline serum glucose and glycosylated hemoglobin A1c (HbA1c) levels were 6.0 mmol/L and 5.8%, respectively. Liver and renal function was normal in this patient. Vascular murmurs were not detected in the neck, chest and abdomen. We also examined hypertensive target organ damage. The carotid femoral pulse wave velocity (cf-PWV) was 8.9 ± 0.8 m/s. The carotid intima media thickness (IMT) was 0.7 mm for both sides and no atheromatous plaques were detected in the carotid arteries. The urinary albumin/creatinine ratio (UACR) was 48.4 mg/g, and the amount of proteinuria was 0.43 g by 24-h urine collection. Echocardiography indicated that the interventricular septum (IVS) thickness was 11 mm, the left ventricular ejection fraction (EF) was 66%, the volume of pericardial effusion was mild and the left ventricular mass index (LVMI) was 84 g/m^2^ according to LVMI (g/m^2^) = LVM/body surface area. The plasma level of N terminal pro-brain natriuretic peptide was 321 pg/ml. Ambulatory blood pressure monitoring (ABPM) showed that the patient had persistently elevated blood pressure (24 h average of 169/102 mmHg, daytime average of 171/103 mmHg, night average of 158/95 mmHg).

### Initial treatment protocol and further examination

**Fig. 2:**
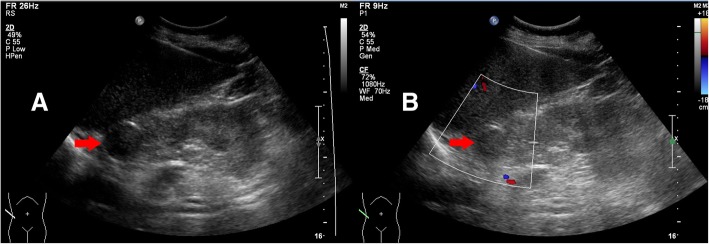
Examination of the adrenal gland by ultrasonography. **a** Two-dimensional ultrasonography: a mass 2.9 cm × 2.8 cm in size with a weak echo was found in the of right side of the adrenal gland. The boundary of the mass was clear. **b** Color doppler ultrasound: no blood flow signal was found in the adrenal gland mass

**Fig. 3:**
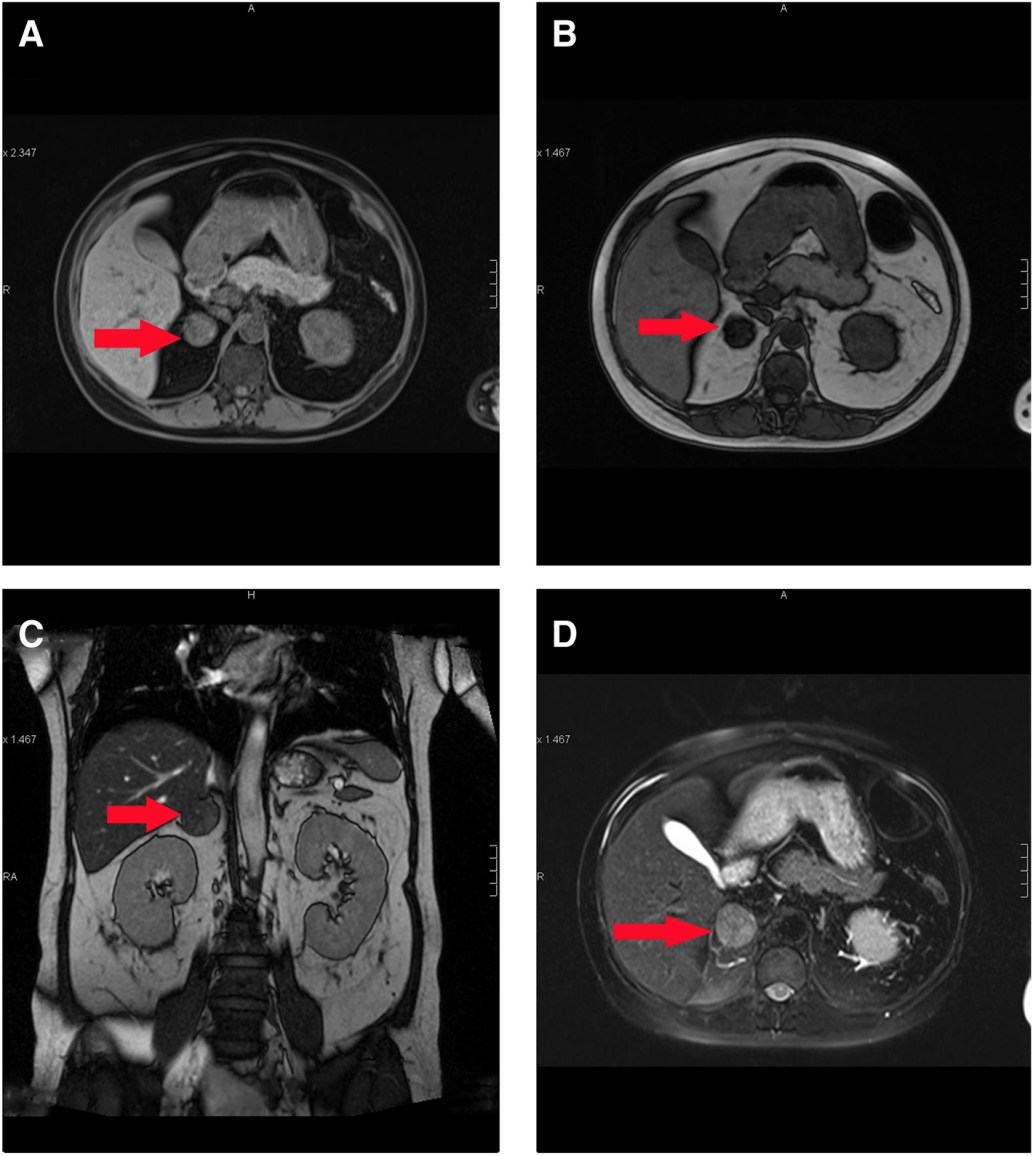
Magnetic Resonance Imaging of the adrenal gland. **a** T1 weighted in phase (T1-IP). **b** T1 weighted opposed phase (T1-OP). **c** T2 weighted. **d** T2 weighted fat suppression (T2-fs). A round-like signal 3.2 cm in diameter was observed in the right side of the adrenal gland. T1W and T2W were both mixed signals for this lesion. The signal of the mass in T1-IP was lower than that in T1-OP, indicating the tumor was rich in lipid and showed pathological characteristics of an adenoma

Methyldopa was prescribed to control the patient’s blood pressure according to the 2013 American College of Obstetricians and Gynecologists guidelines, starting with a small dose (250 mg two times a day) then increasing to a large dose (1 g two times a day). This treatment failed to reduce the patient’s blood pressure to a safe level, and her blood pressure was persistently elevated before the 20th week of gestation. The hypertension was not controlled by 2 g methyldopa per day. In addition, we found the patient had developed hypokalemia (baseline serum potassium was 3.14 moml/L) and preeclampsia in the early second trimester. All of the observed clinical features were not explained by primary or gestational hypertension, so our department measured other endocrine hormones, including catecholamines, hydrocortisone, 24 h UFC, adrenocorticotropic hormone (ATCH) and renin-angiotensin-aldosterone system hormones. Catecholamines, including norepinephrine, epinephrine and dopamine, were normal in both plasma and urine samples. Plasma renin, angiotensin II and aldosterone levels were all in the normal range and the aldosterone/renin ratio (ARR) was 1.94 ng/dl:ng/ml/h. The 24 h UFC was 1684.3 μg/24 h (normal range: 20.26–127.55 μg/24 h). The ATCH level was < 1.00 ng/L in three measurements (normal range: 5–78 ng/L). There was a lack of circadian rhythm for plasma cortisol levels (866.3 nmol/L at 8:00 am, 794.8 nmol/L at 4:00 pm, 806.4 nmol/L at midnight). Ultrasonography demonstrated a mass (2.9 cm × 2.8 cm) with a weak echo and clear boundary in the right side of the adrenal gland. Color doppler ultrasound showed no blood flow signal in the adrenal gland mass (Fig. 2). Magnetic resonance imaging (MRI) without contrast showed a mass of 3.2 cm in diameter in the right adrenal gland, and T1 weighted (T1W) and T2 weighted (T2W) images both provided mixed signals. The signal of mass in the T1W image in phase was lower than that in the T1W opposed phase, which indicated the tumor was rich in lipid and conformed to pathological characteristics of an adenoma (Fig. 3).

### Surgery

**Fig. 4:**
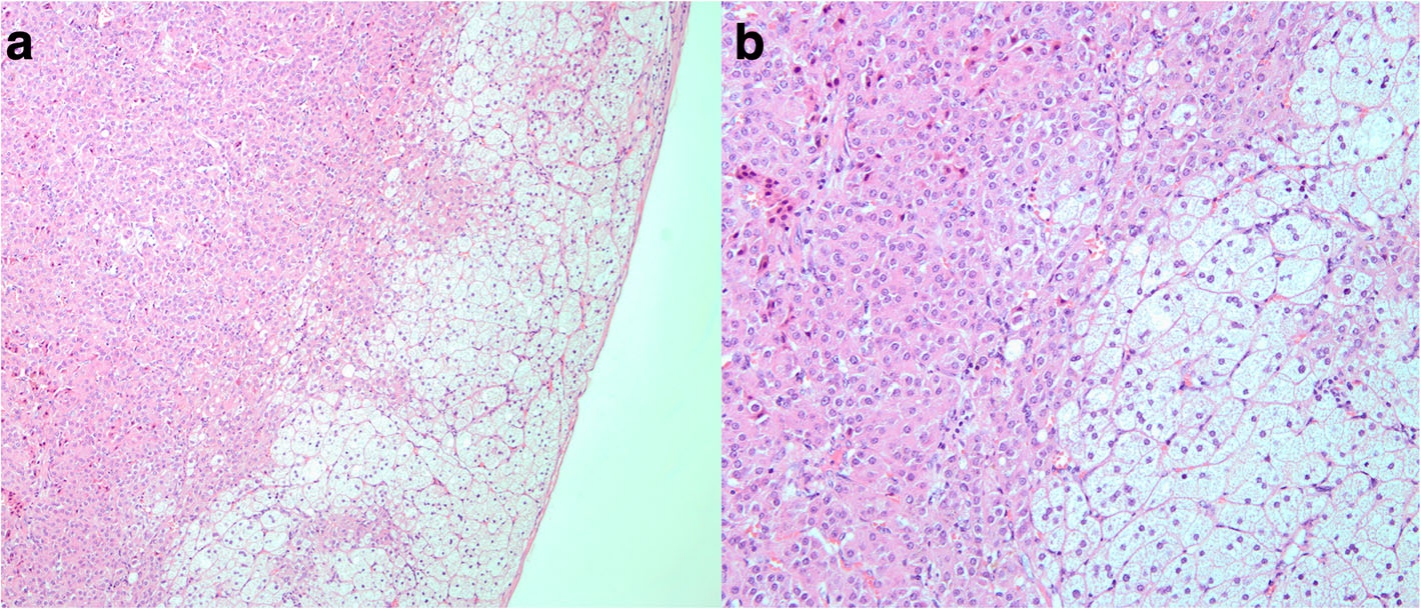
Pathological examination of the adrenal mass after surgery. **a**. Histology (H&E staining, × 100): clear and acidophilic cells were present. The outer membrane of the adrenal cortex was integrated into this pathological picture. **b**. Histology (H&E staining, × 200): clear and acidophilic cells were present. The cells displayed a regular appearance, nuclear heteromorphism was not obvious and pathological mitosis was rare in the images we examined. All of the features indicated that that adrenal mass was most likely an adrenal cortical adenoma

In November 2016, at the 26th week of gestation, laparoscopic adrenalectomy was performed in the right side of the anesthetized patient by a urological surgeon in the West China Hospital. After satisfactory general anesthesia, the pregnant woman was in a left lateral position and a trocar was inserted into the right side of the lumbar region below the costal margin and above the iliac crest. The laparoscope was then inserted into the retroperitoneal region and a 3 cm × 4 cm oval mass with a clear demarcation between the surrounding tissue was discovered in the right side of the adrenal gland. The tumor contained yellow contents and was excised by the urological surgeon. Histopathology indicated an adrenocortical adenoma (pathological mitosis was rare) (Fig. 4).

### Follow up - post surgery

After the surgery, the patient accepted glucocorticoid replacement therapy. Hydrocortisone was administered by intravenous injection at 100 mg daily for 3 days and then 75 mg daily for 10 days. Magnesium sulfate was continuously administered by intravenous infusion to prevent eclampsia and alleviate uterine contraction. Surgical and post-surgical intervention was well tolerated. No adverse and unanticipated events occurred. The patient’s high blood pressure was still present at the beginning of the postoperative period. We prescribed methyldopa (750 mg or 500 mg two times a day) to control the high blood pressure. A month after surgery, the blood pressure decreased gradually and proteinuria was negative by routine urine testing (three measurements). There were no serious adverse events for the pregnant patient and fetus in the remaining second and third trimesters. The patient’s blood pressure was normal at the 32nd week of gestation without antihypertensive medication. Her serum glucose and HbA1c levels were 5.8 mmol/L and 6.1%, respectively. The patient gave birth to a healthy boy by caesarean section at the 40th week of gestation. The baby weighed 3.5 kg, with an apgar score of 8 and 10 at 1 min and 5 min after birth, respectively.

## Discussion and conclusions

Hypertensive disorders of pregnancy (HDP) affect 5–10% of pregnancies worldwide, and are one of the greatest causes of maternal and perinatal morbidity and mortality [8, 9], and include chronic and gestational hypertension, preeclampisa/eclampisa alone and preeclampisa/eclampisa superimposed on chronic hypertension. Chronic hypertension includes primary and secondary hypertensive disorders, which are diagnosed prior to pregnancy or detected before the 20th week of gestation [2, 10]. In contrast, preeclampisa/eclampisa and gestational hypertension are first detected after the 20th gestational week [2, 10]. Secondary hypertension during pregnancy is rare, affecting approximately 0.24% of all pregnancies, but often curable, and includes chronic kidney disease, renovascular hypertension (fibromuscular hyperplasia of the renal arteries and Takayasu’s arteritis), Cushing’s syndrome, pheochromocytoma and primary aldosteronism. Other possible etiologies for secondary hypertension during pregnancy include obstructive sleep apnea, thyroid disease, systemic lupus erythematosus, connective tissue diseases, coarctation of the aorta of pregnant women and medications or supplements used by women during pregnancy [3, 11]. Common causes of secondary hypertension and its clinical features in pregnancy are summarized in Table 1. The symptoms and diseases mentioned above are easily overlooked by obstetricians and general medical doctors in outpatient departments, but can lead to poor fetal and maternal outcomes, as described above [11, 12].

**Table 1:** Summary of secondary hypertension and its clinical features in pregnancy

Causes of secondary hypertension in pregnancy	Prevalence in pregnancy	Clinical features	Clinical Examination
Chronic kidney disease	0.9%	Albuminuria,hypercreatinine,edema,hypertension	Screen albuminuria, serum creatinine and Renal ultrasound
Pheochromocytoma	0.007%	sustained or paroxysmal hypertension, palpitation, cardiomyopathy,impaired glucose tolerance	24-h urinary fractionated metanephrines and catecholamines and plasma fractionated metanephrines; Using abdominal ultrasound or a non-contrast MRI for localization of tumor
Primary aldosteronism	0.6–0.8%	increased secretion of aldosterone, low plasma renin activity, and hypertension, hypokalemia.	increased Plasma aldosterone concentrations and suppressed plasma renin activity; Abdominal ultrasonography or MRI scan can be performed if there is high clinical suspicion for an adrenal mass.
Renovascular hypertension (including fibromuscular dysplasia and Takayasu’s arteritis)	–	Hypertension at a young age(<35 years) with abdominal vascular murmurs	ultrasound examination, MRI, angiography
Cushing’s syndrome	–	Weight gain, striae, Moon Face,facial acne,impaired glucose tolerance	24 h urine free cortisol elevation, loss of circadian fluctuation in cortisol; Using abdominal ultrasound or a non-contrast MRI for localization of tumor
obstructive sleep apnea	4.9%	Snoring, hypertension	Polysomnography
Other uncommon cause	Thyroid disease, maternal coarctation of aorta, systemic lupus erythematosus and other connective tissue disease		

In this case report, a pregnant woman with Cushing’s syndrome presented with severe hypertension. It is difficult for Cushing’s syndrome patients to become pregnant because of abnormal menses and the difficulty conceiving [13], as well as the increased risk of maternal and fetal complications from the high serum androgen and cortisol levels. The first case-report of pregnancy with Cushing’s syndrome was by Hunt and McConahey in 1953 [4, 14], and currently there are nearly 200 cases of Cushing’s syndrome during pregnancy reported in the literature [5]. Despite the low prevalence of pregnancy with Cushing’s syndrome, considering its association with poor pregnancy outcomes, more attention is required for the clinical diagnosis and management of this disease. The main cause of Cushing’s syndrome during pregnancy is adrenal adenoma, accounting for 40–60% cases, followed by pituitary adenoma and adrenocortical carcinoma [7, 13, 15].

It is difficult to diagnose Cushing’s syndrome in pregnant women, because pregnancy can influence both the maternal hypothalamic-pituitary-adrenal axis and the renin-angiotensin-aldosterone system and the specific criteria to assess hormone levels are lacking for pregnancy with hypercortisolism [16–19]. The physiological increase of corticotropin-releasing hormone (CRH), ACTH and corticosteroid-binding globulin (CBG) form the placenta during pregnancy causes a slight elevation of cortisol levels (serum, salivary and urinary) [16]. Biochemical diagnosis for Cushing syndrome is complicated with respect to non-pregnant women. The plasma level of cortisol starts to increase from the first trimester and lasts for the remaining pregnant period, which can rise 2–3 times above normal (non-pregnant levels) in pregnant women [19]. Firstly, elevated concentration of cortisol transport protein, i.e. Corticosteroid Binding Globulin (CBG), leads to 2–3-fold elevation of plasma cortisol level [20]. Secondly, the CRH and ACTH secreted by the placenta in pregnancy elevates the plasma cortisol level as well [21]. Nonetheless, the circadian rhythm of cortisol may be preserved during pregnancy. UFC provides an integrated assessment of cortisol secretion over a 24-h period. UFC increases 1.4 to 1.6 fold in the second and third trimester, respectively [18]. A significant elevation of UFC (at least 2–3 fold higher than the upper limit of normal value) is a biomarker for Cushing’s syndrome in pregnancy according to Endocrine Society guidelines [22]. According to Yaneva et al. the sensitivity of UFC measurement is 45 to 71% [23, 24]. Salivary cortisol reflects the free fraction of total serum cortisol representing the unbound, biologically active form of serum cortisol and is not influenced by binding protein [25]. Ambroziak et al. have found that the salivary cortisol did not significantly change in pregnancy and suggested that reference values for salivary cortisol established for a healthy adult population could be used for pregnant women in the initial diagnostic testing for Cushing’s syndrome [18, 26, 27]. But, The value of using salivary cortisol for the diagnosis of Cushing’s syndrome in pregnancy remains ambiguous [13]. More than 80% of the 1 mg dexamethasone suppression tests (DSTs) have false positive results in normal pregnant women, caused by an altered baseline of the hypothalamic-pituitary-adrenal axis during gestation, it is not recommended to use the overnight 1 mg DST to diagnose Cushing’s syndrome in pregnancy [22, 28, 29]. To summarize, most literature reviews recommend utilizing the UFC level at more than three times the upper normal limit to evaluate for Cushing’s syndrome in pregnancy during the second or third trimesters [22]. Some studies suggested that the circadian rhythm of blood cortisol levels could be used to diagnose Cushing’s syndrome in pregnant women [30]. Therefore, more attention should be given to the UFC level and circadian rhythm of blood cortisol in diagnosing cases of Cushing’s disease during pregnancy.

Following a clear diagnosis, it is important to determine the etiology of Cushing’s syndrome in the patient. Plasma ATCH and a high-dose DST may be used to identify ACTH-dependent or non-ACTH dependent Cushing’s syndrome, and may suggest further examination for the presence of an adrenal tumor [16, 31]. Adrenal imaging by ultrasound or nongadolinium-contrasted MRI are routinely used to locate adrenal tumors [6, 13, 15, 16, 32].

Pregnant women with Cushing’s syndrome have a higher incidence of poor gestational outcomes, including increased risks of gestational diabetes mellitus, hypertension and preeclampsia [5]. In 1987, Bevan et al. investigated the timing of treatment for Cushing’s syndrome during pregnancy [33]. This previous study of 41 pregnant women found a reduced rate of fetal loss and an increased rate of successful pregnancy in the group treated during pregnancy compared with the group treated after pregnancy [33]. Considering the above findings, positive outcomes can be expected from the thorough clinical management of pregnant women with Cushing’s syndrome.

Management of patients with Cushing’s syndrome caused by an adrenal adenoma includes laparoscopic surgical resection and medical therapy. Similar to the treatment of non-pregnant women, surgery is the first choice to treat Cushing’s syndrome due to adrenal adenoma during pregnancy, considering the potential harmful and teratogenic effects upon the baby of alternative radiotherapy and medical treatments [15, 16]. The optimal time for adrenal adenoma surgery is during the secondary trimester, because it was relatively safe and increased live birth rates, and had no impact upon the rate of premature birth or intrauterine growth restriction [34, 35]. Other reasons for selecting the optimal time of adrenal adenoma surgery during the secondary trimester should also be considered. First, the risk of spontaneous miscarriages is reduced in the second compared with the first trimester. Second, the uterus is not large enough to impede intra-abdominal procedures in the second compared with the third trimester. Other considerations include the surgeon’s expertise, severity of the disease, and the patient’s preference. The most successful surgery for cases of adrenal adenoma in pregnancy involve laparoscopic surgical resection. According to a review by Lal and Duh, laparoscopic adrenalectomy is objectively safe during pregnancy [36]. Sammour et al. reported that adrenal adenoma surgery during pregnancy reduced both maternal and perinatal morbidity, and laparoscopy was the preferred surgical approach in 23 patients with Cushing’s syndrome due to adrenal adenoma [30]. Medical treatment may also consider the use of metyrapone, a steroidogenic inhibitor of the conversion of 11-deoxycortisol to cortisol and the first choice to effectively control hypercortisolism. However, side-effects of metyrapone include increased levels of mineralocorticoid precursors, which can elevate blood pressure and increase the risk of preeclampsia [6]. Other inhibitors of steroidogenesis, such as ketoconazole, mitotane and aminoglutethimide, should not be prescribed during pregnancy, because of the lack of investigation regarding safety during pregnancy, and potential teratogenicity and fetal masculinization induced by these drugs during pregnancy [13, 37]. When surgical therapy is contraindicated, or as an interim therapy before adrenal adenoma surgery, metyrapone may be used to treat pregnant patients exhibiting serious hypercortisolism [13].

Considering the findings of this case report, obstetricians should not overlook secondary hypertension in pregnancy. For pregnant women with high blood pressure that fails to be controlled by relatively high doses of antihypertensive drugs, obstetricians should seek help from cardiologists to exclude common causes of secondary hypertension. Analysis of the UFC level and circadian rhythm of blood cortisol provided a reasonable strategy to diagnosis Cushing’s syndrome in a pregnant patient. Laparoscopic surgical resection in the second trimester provides a reasonable approach to treat pregnant patients exhibiting Cushing’s syndrome caused by an adrenal adenoma. The treatment of such cases should also consider the surgeon’s expertise, severity of the disease, and the patient’s preference.

## Abbreviations

ABPM: Ambulatory blood pressure monitoring
ARR: Aldosterone renin ratio
ATCH: Adrenocorticotropic hormone
cf-PWV: carotid femoral pulse wave velocity
CS: Cushing’s syndrome
DST: Dexamethasone suppression test
EF: Ejection fraction
HDP: Hypertensive disorders of pregnancy
IMT: Intima media thickness
IVS: Interventricular septum
LVMI: Left ventricular mass index
MRI: Magnetic resonance imaging
UACR: Urinary albumin/creatinine ratio
UFC: Urinary free cortisol
